# Genetic Variability in Phosphorus Responses of Rice Root Phenotypes

**DOI:** 10.1186/s12284-016-0102-9

**Published:** 2016-06-13

**Authors:** Phanchita Vejchasarn, Jonathan P. Lynch, Kathleen M. Brown

**Affiliations:** Department of Plant Science, Penn State University, University Park, PA 16802 USA; Present address: Ubonratchathani Rice Research Center, Ubon Ratchathani, USA

**Keywords:** Aerenchyma, Root anatomy, Lateral roots, *Oryza sativa*, Phosphorus, Root hairs, Stele, Xylem

## Abstract

**Background:**

Low phosphorus availability is a major factor limiting rice productivity. Since root traits determine phosphorus acquisition efficiency, they are logical selection targets for breeding rice with higher productivity in low phosphorus soils. Before using these traits for breeding, it is necessary to identify genetic variation and to assess the plasticity of each trait in response to the environment. In this study, we measured phenotypic variation and effect of phosphorus deficiency on root architectural, morphological and anatomical traits in 15 rice (*Oryza sativa*) genotypes. Rice plants were grown with diffusion-limited phosphorus using solid-phase buffered phosphorus to mimic realistic phosphorus availability conditions.

**Results:**

Shoot dry weight, tiller number, plant height, number of nodal roots and shoot phosphorus content were reduced under low phosphorus availability. Phosphorus deficiency significantly reduced large lateral root density and small and large lateral root length in all genotypes, though the degree of plasticity and relative allocation of root length between the two root classes varied among genotypes. Root hair length and density increased in all genotypes in response to low phosphorus. Nodal root cross-sectional area was significantly less under low phosphorus availability, and reduced cortical area was disproportionately responsible for this decline. Phosphorus deficiency caused a 20 % increase in the percent cortical area converted to aerenchyma. Total stele area and meta-xylem vessel area responses to low phosphorus differed significantly among genotypes. Phosphorus treatment did not significantly affect theoretical water conductance overall, but increased or reduced it in a few genotypes. All genotypes had restricted water conductance at the base of the nodal root compared to other positions along the root axis.

**Conclusions:**

There was substantial genetic variation for all root traits investigated. Low phosphorus availability significantly affected most traits, often to an extent that varied with the genotype. With the exception of stele and meta-xylem vessel area, root responses to low phosphorus were in the same direction for all genotypes tested. Therefore, phenotypic evaluations conducted with adequate fertility should be useful for genetic mapping studies and identifying potential sources of trait variation, but these should be confirmed in low-phosphorus environments.

**Electronic supplementary material:**

The online version of this article (doi:10.1186/s12284-016-0102-9) contains supplementary material, which is available to authorized users.

## Background

The on-going increase of human population in developing countries requires an increase in crop yields to meet the growing demands for food. In many of these countries, however, agricultural productivity is limited by soil infertility. Rice is the most important staple food for more than half of the world’s population and an important model for cereal crops. Although phosphorus (P) is essential for plant growth, it is one of the least available nutrients in many agroecosystems, principally because P is bound to soil chemical and biological components that make it unavailable to growing plants (Richardson et al. 2009). In modern farming systems, P fertilization is used to remedy P deficiency. However, rock phosphate reserves are limited, fertilizers are costly for subsistence farmers, and phosphate use is inefficient because of immobilization by the soil (Cordell et al. 2009). Improving P efficiency of crops would be an important contribution to sustainability of agroecosystems (Richardson et al. 2011), both in developing countries where fertilizer use is negligible and in developed countries where excess P fertilization is responsible for environmental degradation (Cordell et al. 2009).

P deficiency is considered a major limiting factor for rice production, especially in upland and rainfed-lowland production systems (Kirk et al. 1998; Ismail et al. 2007) and almost 50 % of rice soils are considered P deficient (Ismail et al. 2007). A key aspect of improving crop performance in low-P soils is improving P acquisition efficiency via improved root traits (Lynch and Brown 2008; Richardson et al. 2011; Rose et al. 2012). Since P is diffusion-limited and depletion zones develop around existing roots, continuous exploration of soil is important for P acquisition. Root systems that can explore relevant soil domains at low metabolic cost are particularly important, since roots depend on and compete with shoots for fixed carbon (Lynch and Ho 2005; Lynch 2014). A number of root traits have been shown to be important for P acquisition in crops, including root hairs (Fohse et al. 1991; Gahoonia et al. 1997; Gahoonia and Nielsen 1998, 2003; Bates and Lynch 2000; Vandamme et al. 2013), traits affecting topsoil exploration such as axial root angle (Lynch and Brown 2001), and elongation of lateral roots with high specific root length (Zhu and Lynch 2004). Similar strategies are likely to be useful in developing rice genotypes with better P acquisition efficiency.

Anatomical traits affect P efficiency via their effects on root metabolic cost, e.g. enhanced root cortical aerenchyma (RCA) formation reduces root respiration and the metabolic cost of soil exploration in maize subject to drought and low nitrogen (Zhu et al. 2010; Saengwilai et al. 2014). The functional-structural model *SimRoot* predicts that more RCA would improve P efficiency in maize, substantially improving growth and reducing critical P levels by 12 % (Postma and Lynch 2011). Despite the fact that rice has substantially greater RCA formation than maize, genotypic variation in rice RCA could affect metabolic cost by a similar mechanism. Increased specific root length, a phenomenon often observed under low nutrient treatments (Hill et al. 2006; Fernandez and Rubio 2015), is also predicted to reduce the cost of soil exploration (Chimungu and Lynch 2015). Changes in specific root length could be achieved by reduced secondary growth in dicots, or by various anatomical changes in monocots, such as fewer cortical cells or a smaller stele. Cortical cell file number, which is correlated with the number of cortical cells, has been shown to improve drought tolerance of maize by reducing root respiration, increasing rooting depth and thereby improving water capture (Chimungu et al. 2014). In rice, anatomical traits such as root diameter and xylem vessel size have previously been targeted for their potential to improve drought resistance (Clark et al. 2008; Henry et al. 2012), but could also contribute to root efficiency, i.e. P uptake per unit root size (Wissuwa 2005), under low P conditions.

Genotypic variation in root traits provides a potential genetic resource for plant breeders. Genetic variation has been demonstrated and QTL mapped for many root traits in rice, including thickness, rooting depth, stele area, xylem vessel size and aerenchyma formation (Coudert et al. 2010; Gowda et al. 2011). However, genetic variation for the effect of low P on these traits has not been investigated. Many root traits are plastic, i.e. the phenotype is altered by environmental factors including P availability. Plasticity itself has a genetic component, e.g. QTL have been identified for plasticity of lateral root length and number (Zhu et al. 2005a) and root hair length (Zhu et al. 2005b) in maize seedlings grown under high and low P. In rice, QTL have been detected for plasticity of lateral root (Kano et al. 2011) and aerenchyma development (Niones et al. 2013) in response to drought, and for seminal root elongation in response to low N and low P (Ogawa et al. 2014). Since genotypes vary for both phenotypic expression and for plasticity in response to environmental factors such as P availability, it is important to assess both genetic variation and plasticity of traits relevant to P acquisition efficiency before exploiting these traits in a breeding program.

In this study, genetic variation and plasticity in response to low P are assessed for architectural, morphological and anatomical traits in 15 rice (*Oryza sativa* L.) genotypes. Natural genetic variation in plasticity of these traits in response to P availability has not been previously reported.

## Results

Genetic variation in root traits was examined in 15 genotypes of cultivated rice (Table 1). We also examined variation in root hairs and anatomical traits at four axial positions along the nodal roots.

**Table 1:** Rice cultivars (*Oryza sativa*) used for evaluation of phosphorus effects on root phenotypes

Cultivar Name	Country of origin	Sub-population	Varietal group	GSOR^a^ ID
IR 64	Philippines	*indica*	*Indica*	GSOR#312010
Pokkali	Sri-Lanka	*indica*	*Indica*	GSOR#312020
Patnai 23	India	*indica*	*Indica*	GSOR#301118
Leung Pratew	Thailand	*indica*	*Indica*	GSOR#301094
Kasalath	India	*aus*	*Indica*	GSOR#301077
Jhona 349	India	*aus*	*Indica*	GSOR#301071
Dular	India	*aus*	*Indica*	GSOR#301044
Aichi Asahi	Japan	*temperate japonica*	*Japonica*	GSOR#301002
Nipponbare	Japan	*temperate japonica*	*Japonica*	GSOR#301164
BicoBranco	Brazil	*aromatic*	*Japonica*	GSOR#301014
Basmati	Pakistan	*aromatic*	*Japonica*	GSOR#301011
Dom-sofid	Iran	*aromatic*	*Japonica*	GSOR#301042
Moroberekan	Guinea	*tropical japonica*	*Japonica*	GSOR#301100
Cocodrie	United States	*tropical japonica*	*Japonica*	GSOR#301379
Azucena	Philippines	*tropical japonica*	*Japonica*	GSOR#301165

^a^ USDA genetic stock identification number

### Axial Distribution of Root Hairs

There were significant differences in average root hair length and root hair density among axial positions and genotypes (Table 2). As expected, root hairs near the root tip were not fully developed, and both density and length were lower at the 5 cm position than at the other sampling positions. Among all axial positions and genotypes, root hair length ranged from 0.18 mm to 0.32 mm and root hair density ranged from 183 hairs · mm^−2^ to 238 hairs · mm^−2^. The position for root hair sampling for subsequent experiments was 5–10 cm from the root tip, based on maximal root hair development at that position.

**Table 2:** The effects of axial position and genotype on root hair traits

		Root hair length (mm)		Root hair density (hairs · mm^−2^)	
	d.f.	F	P	F	P
Axial Position (AP)	2	20.959	<0.001	11.523	<0.001
Genotype	14	22.129	<0.001	2.585	0.003
AP*Genotype	28	3.015	<0.001	0.399	0.996
AP		Mean	SE	Mean	SE
Tip (5 cm)		0.225 a	0.005	208.5 a	2.2
Middle (10 cm)		0.264 b	0.005	221.1 b	2.4
Base (15 cm)		0.263 b	0.005	222.4 b	2.2

Analysis of variance, means and standard errors (SE) are shown for root hair traits measured at different axial positions: tip (0–5 cm), middle (5–10 cm), and base (10–15 cm) from the root apex. Different letters indicate significant differences among axial positions by the least significant difference (LSD) test (*P* < 0.05)

### Axial Distribution of Root Anatomical Phenes

**Fig. 1:**
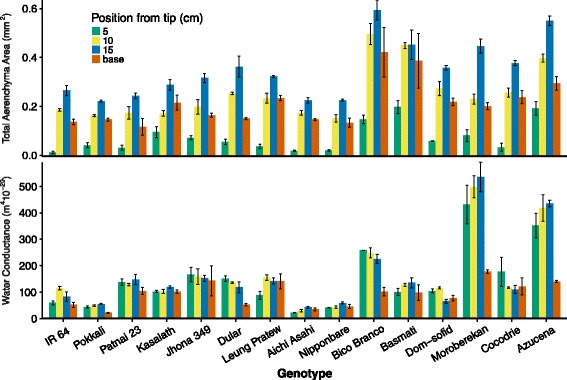
Distribution of aerenchyma area and water conductance among genotypes and sampling positions. Samples were taken close to the base and at 5, 10 or 15 cm from a nodal root tip. Values shown are means of three replications. See Table 3 for statistical analyses

RCA typically develops in mature segments of the root, beyond the elongation zone (Justin and Armstrong 1987; Kawai et al. 1998; Burton et al. 2012a). Since the position of maximal RCA formation could vary among genotypes, we examined the distribution of RCA along the axis of nodal roots. RCA formation was first visible 4–5 cm from the root tip and was fully developed by 15 cm from the root tip (Table 3). Axial position had a strong and significant effect on the absolute area (AA) and percent of aerenchyma (%AA) (Fig. 1 and Table 3). In all accessions, the greatest mean AA was found 15 cm from the root tip (Fig. 1). Therefore, we selected 15 cm from the root tip as the sampling position for investigating the effects of low P on RCA and other root anatomical traits. Despite the overall trend toward greater RCA (both AA and %AA) with distance from the root tip, the amount of RCA was significantly less in the basal region (1–2 cm below the root-shoot junction), which is consistent with studies in maize (Bouranis et al. 2006; Siyiannis et al. 2011; Burton et al. 2012a).

**Table 3:** The effects of axial position and genotype on anatomical traits

	d.f.	TCA (mm^2^)		AA (mm^2^)		%AA		LCA (mm^2^)	
		F	P	F	P	F	P	F	P
Axial Position (AP)	3	1.450	0.230	56.266	<0.001	188.553	<0.001	14.923	<0.001
Genotype	14	29.891	<0.001	6.997	<0.001	0.923	0.535	10.088	<0.001
AP*Genotype	42	7.224	<0.001	2.181	0.001	3.138	<0.001	7.947	<0.001
Axial Positions		Mean	SE	Mean	SE	Mean	SE	Mean	SE
5 cm		0.768a	0.038	0.072a	0.063	8.61a	0.92	0.696c	0.032
10 cm		0.813a	0.042	0.253b	0.109	30.88b	0.84	0.559b	0.029
15 cm		0.792a	0.045	0.349c	0.120	44.87c	0.93	0.443a	0.030
base		0.706a	0.026	0.213b	0.108	29.72b	1.53	0.493ab	0.020
		TSA (mm^2^)		MXV		MXA (mm^2^)		WC (m^4^ · 10^−20^)	
		F	P	F	P	F	P	F	P
AP	3	0.807	0.492	3.489	0.017	4.833	0.003	3.459	0.018
Genotype	14	47.401	<0.001	26.655	<0.001	21.634	<0.001	32.137	<0.001
AP*Genotype	42	4.167	<0.001	7.267	<0.001	3.619	<0.001	5.683	<0.001
Axial Positions		Mean	SE	Mean	SE	Mean	SE	Mean	SE
5 cm		0.072a	0.004	5.02a	0.178	0.0015b	0.000077	149.18b	18.075
10 cm		0.081a	0.005	5.20a	0.200	0.0016b	0.000081	162.92b	19.669
15 cm		0.076a	0.004	5.36ab	0.163	0.0015b	0.000080	161.78b	20.938
base		0.074a	0.003	5.82b	0.191	0.0012a	0.000058	94.27a	8.091

Analysis of variance, means and standard errors (SE) are shown for total cortical area (TCA), aerenchyma area (AA), percent aerenchyma (%AA), living cortical area (LCA), total stele area (TSA), number of late-metaxylem vessels (MXV), median area of the late-metaxylem vessels (MXA), and water conductance (WC) measured at 5, 10 and 15 cm from the root apex and at 1 cm from the root base. Letters indicate significant differences among axial positions by the least significant difference (LSD) test (*P* < 0.05)

Total stele area (TSA), number of late metaxylem vessels, and median metaxylem vessel area (MXA) varied somewhat with distance from the root tip, though the direction and extent of variation depended on the genotype (Table 3, Additional file 1: Figure S1). TSA and MXA were slightly correlated (*r* = 0.44). Two of the *tropical japonica* lines, Moroberekan and Azucena, had the largest metaxylem vessels and larger-than average stele areas. When water conductance was calculated based on the size and number of late metaxylem vessels, the Moroberekan and Azucena had substantially greater water conductance than the other genotypes tested (Fig. 1). Across all genotypes, water conductance was less at the base of the crown root than at the other sampling positions. Cocodrie had substantially greater water conductance at the sampling position closest to the root tip compared to other sampling positions, but in general, water conductance was equal or less at the 5 cm position compared to 10 and 15 cm.

### Responses to Low P: Growth

**Fig. 2:**
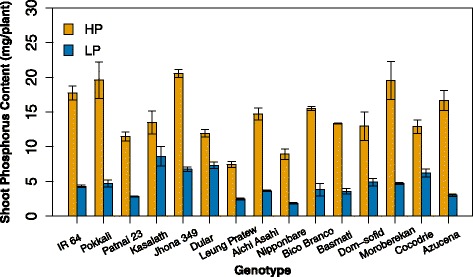
P effect on shoot P content. Plants were harvested at V8 for P analysis. Values shown are means of three replications ± SE. See Table 4 for statistical analysis

**Fig. 3:**
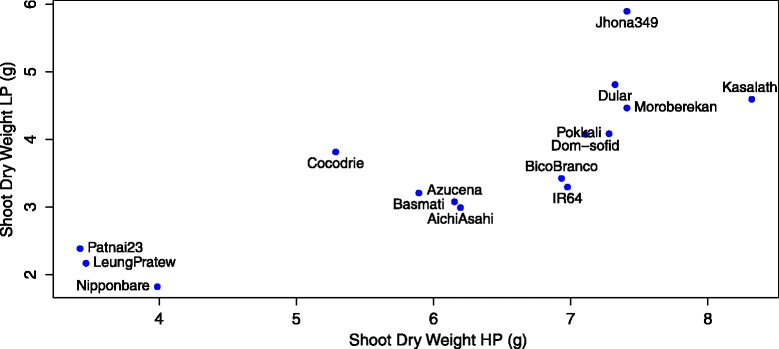
Relationship of shoot dry weights between high P and low P treatments. Genotypes in the upper right quadrant were vigorous under both P levels

In this study, rice plants were cultivated in diffusion-limited P using the solid-phase buffered Al-P method (Lynch et al. 1990), which produces realistic P availability regimes in the growth medium. Low-P treatments were effective in producing P stress, as shown by reduced shoot biomass, tiller number, plant height and shoot P content (Fig. 2, Table 4). Low-P treatment reduced shoot biomass and tiller number by 42 and 41 %, respectively and reduced shoot phosphorus content by 68 % (Table 4). Differences among genotypes were observed for all growth-related variables (Table 4). Additionally, significant genotype x P treatment interactions were observed for shoot biomass, number of tillers and shoot phosphorus content. Since low P reduced shoot biomass but did not significantly affect root biomass, root to shoot ratio was increased under low P (Table 4). Shoot dry weights under low P were plotted against those under sufficient P to identify lines with high vigor under both P treatments (Fig. 3). There was a wide variation in vigor among genotypes, with the three *aus* genotypes showing the strongest ability to maintain shoot growth under low phosphorus availability (33 % reduction in shoot dry weight with low P in *aus* genotypes vs. 39–48 % reductions in the other subpopulations). Although most growth-related variables were also affected by varietal group, there were no interactions of varietal group with P treatment (Additional file 2: Table S2).

**Table 4:** The effects of genotype and phosphorus treatment on shoot and root morphological traits

	d.f.	Shoot biomass (g · plant^−1^)		Plant height (cm)		Number of tillers (plant^−1^)		Root dry weight (g · plant^−1^)	
		F	P	F	P	F	P	F	P
Genotype (G)	14	3.807	<0.001	25.907	<0.001	5.285	<0.001	5.684	<0.001
Treatment (P)	1	82.269	<0.001	9.979	0.002	33.657	<0.001	1.874	0.175
G * P	14	4.808	<0.001	0.910	0.553	7.660	<0.001	1.630	0.097
Treatments		Mean	SE	Mean	SE	Mean	SE	Mean	SE
HP		6.21	0.232	100.44	2.013	5.69	0.361	1.581	0.109
LP		3.61	0.169	91.47	2.006	3.37	0.169	1.343	0.135
Mean		4.91	0.199	95.96	1.491	4.53	0.233	1.462	0.087
	d.f	Root hair length (mm)		Root hair density (hairs · mm^−2^)		Nodal root number (plant^−1^)		Root:shoot ratio	
		F	P	F	P	F	P	F	P
Genotype (G)	14	7.202	<0.001	1.849	0.047	2.239	0.013	2.272	0.012
Treatment (P)	1	26.228	<0.001	47.354	<0.001	110.074	<0.001	14.060	<0.001
G * P	14	0.341	0.985	0.542	0.898	2.696	0.004	1.293	0.239
Treatments		Mean	SE	Mean	SE	Mean	SE	Mean	SE
HP		0.214	0.005	213.958	2.875	88.67	2.32	0.251	0.012
LP		0.251	0.005	238.051	1.998	58.71	1.66	0.355	0.025
Mean		0.232	0.004	226.005	2.159	73.69	2.13	0.303	0.015
	d.f	Small lateral root length (cm)		Large lateral root length (cm)		Lateral root density		Shoot P content (mg · plant^−1^)	
		F	P	F	P	F	P	F	P
Genotype (G)	14	4.491	<0.001	2.596	0.004	1.70	0.073	0.848	0.616
Treatment (P)	1	37.796	<0.001	67.354	<0.001	85.971	<0.001	142.801	<0.001
G * P	14	2.655	0.004	1.291	0.240	1.35	0.207	2.198	0.018
Treatments		Mean	SE	Mean	SE	Mean	SE	Mean	SE
HP		94.098	5.708	236.289	8.876	48.98	1.12	14.45	0.752
LP		48.537	4.727	140.640	7.553	36.49	0.75	4.59	0.341
Mean		71.318	4.405	188.465	7.699	42.73	0.94	9.52	0.664

Analysis of variance (ANOVA), means and standard error (SE) values are shown for 15 genotypes grown with high (100 μM; HP) or low phosphorus (2 μM; LP) treatments

### Architectural Responses to Low P: Nodal Root Number and Lateral Root Branching

**Fig. 4:**
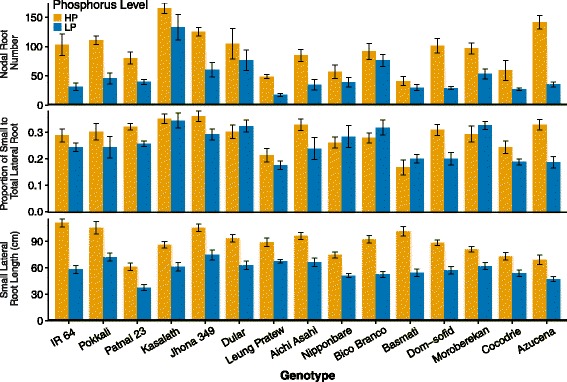
Effect of genotype and P treatment on root branching. Values shown are means of three replications ± SE for nodal root number, small lateral root length and the proportion of total lateral root length as small lateral roots. See Table 4 for statistical analyses

Nodal root number varied from 61 to 111 with adequate P and was reduced by 23–47 % under low P availability (Fig. 4). IR64 had the greatest number of nodal roots with adequate P and the greatest reduction (47 %) under low P. Lengths of small and large lateral roots were significantly affected by genotype and P treatment (Fig. 4, Table 4). Low P availability resulted in a reduction in small and large lateral root length in all genotypes by and average of 48 % and 41 %, respectively (Table 4). There was a significant genotype x P interaction for small, but not large, lateral root length (Fig. 4, Table 4); reductions in small lateral root length under low P ranged from 16 % in Bico Branco to 75 % in Azucena. With low P treatment, the length of small lateral roots was reduced to a greater extent than large lateral roots in most genotypes, even though large lateral roots represented a much greater investment (small lateral roots are defined as <0.1 mm diameter). The proportion of small to total lateral root length was reduced from 0 to 43 % with low P availability, depending on the genotype, with the *tropical japonica* genotype Azucena having the greatest reduction.

Lateral root density was affected by genotype only at *P* = 0.07 (Table 4) and there was no interaction of phosphorus treatment with genotype (Table 4). Low P reduced lateral root density by 25 % on average, with Aichi Asahi showing the smallest reduction (11 %) and Dular showing the largest reduction (33 %). For the root architecture traits, varietal group had a significant effect only on small lateral root length, and there were no significant interactions of varietal group and P treatment (Additional file 2: Table S2).

Since low P treatment results in less plant growth, which can affect the allocation of resources between roots and shoots and among different root classes, we determined the allometric relationships of root traits with shoot dry weight. The scaling exponent (α) for small lateral root length calculated for all genotypes was very similar for high and low P treatments and close to the expected isometric value (0.33, Table 5), i.e. the increase in small lateral root length was proportional to the increase in shoot dry weight. For large lateral root length, the scaling exponent was greater for high P than for low P (0.68 vs. 0.47) and both were greater than the isometric value, indicating a disproportionately larger increase in large lateral root length relative shoot dry weight, especially in high-P plants (Table 5). Lateral root density also scaled somewhat greater than expected under high P.

**Table 5:** Allometric analyses of root traits in plants grown with high or low Pk

	High P		Low P	
Variable name	R^2^	α	R^2^	α
Small lateral root length^a^	0.287*	0.361	0.241*	0.347
Large lateral root length^a^	0.345*	0.682	0.177*	0.471
Lateral root density^a^	0.063	0.503	0.013	0.323
Nodal root number^a^	0.309	0.929	0.218	0.916
TCA^2^	0.996*	0.988	0.992*	0.982
TSA^2^	0.759*	0.753	0.616*	0.580
AA^2^	0.699*	0.690	0.657*	0.634
%AA^b^	<0.001	0.009	0.001	0.047
MXV^b^	0.202*	0.579	0.225*	0.641
MXA^b^	0.001	0.021	0.001	0.029
WCP^b^	0.511*	0.271	0.530*	0.307

The ln of each trait was run against the ln of total DW (lateral roots) or ln of RXSA (anatomical traits). The allometric scaling exponent α was calculated from the slope of the linear relationship. Significant regressions are indicated by *

^a^Against the ln of total plant dry weight

^b^Against the ln root cross-sectional area

### Responses to Low P: Root Hair Characteristics

**Fig. 5:**
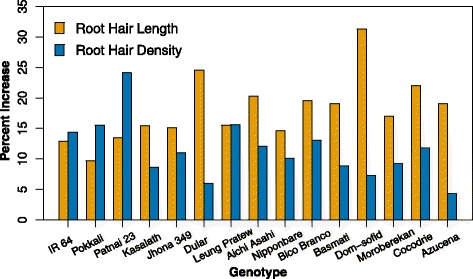
Increases in root hair length and density with low P treatment. Percent changes were calculated from three replications per genotype and treatment

Root hair length and density were significantly affected by genotype and P treatment (Table 4) and by varietal group (Additional file 2: Table S2). Azucena had the shortest and densest hairs of all the genotypes at both P levels. Low P availability increased root hair length by an average of 15 %, with a genotypic range of 9.7–31.3 %, and low P increased root hair density by an average of 10 % with a genotypic range of 4.4–24.1 % (Fig. 5). Root hairs of *Japonica* genotypes were shorter and denser than those of *Indica* genotypes (Additional file 2: Table S2). Overall, genotypes produced similar increases in root hair length and density in response to low P, i.e. there were no significant genotype x P interactions (Table 4) or varietal group by P interactions (Additional file 2: Table S2).

### Responses to Low P: Root Anatomical Characteristics

**Fig. 6:**
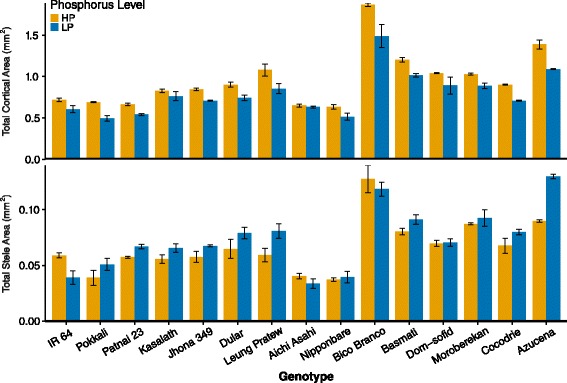
Effects of genotype and phosphorus treatment on total cortical area and total stele area. Values shown are means of three replications ± SE. See Table 6 for statistical analyses

The total root cross-sectional area (RXSA), total cortical area (TCA), stele area (TSA) and TCA to RXSA ratio varied greatly among genotypes (Fig. 6, Table 6). Low P reduced RXSA by an average of 15 % over all genotypes (Table 6). RXSA was highly correlated with TCA (*r* = 0.998, *P* < 0.01), which was not surprising since mean TCA comprised 92 % of the total RXSA (see overall means, Table 6). TSA was also highly correlated with RXSA (*r* = 0.853, *P* < 0.01). Note that in these analyses, RXSA is the sum of TSA and TCA, since *RootScan* does not detect epidermal and endodermal cell layers. Low P treatments affected the components of RXSA differently. Total cortical area (TCA) was reduced by low P treatment for all genotypes (mean reduction 16.8 %), with Pokkali exhibiting the greatest reduction (28 %) and Aichi Asahi the least (2.6 %), which was similar to the effects of low P on RXSA (Fig. 6). The effects of low P on TSA varied from more than 33 % reduction in IR64 to more than 44 % increase in Azucena (Fig. 6). As a result, there was also genetic variation for the ratio of TCA to RXSA (Table 6). All of these variables except TCA:RXSA showed significant genotype by P interactions. *Japonica* genotypes had thicker roots with larger RXSA, TSA, TCA and LCA, but varietal group did not significantly interact with P treatment (Additional file 3: Table S3), indicating that the genotype * P interactions were not explained by varietal group classification.

**Table 6:** The effects of genotype and phosphorus treatment on anatomical traits

	d.f.	RXSA (mm^2^)		TSA (mm^2^)		TCA (mm^2^)		TCA:RXSA Ratio		LCA (mm^2^)	
		F	P	F	P	F	P	F	P	F	P
Genotype (G)	14	44.001	<0.001	27.366	<0.001	34.778	<0.001	2.695	0.003	14.034	<0.001
Treatment (P)	1	5.564	0.021	1.886	0.173	6.996	0.010	21.756	<0.001	17.178	<0.001
G * P	14	1.954	0.038	3.727	<0.001	2.041	0.029	1.429	0.168	3.001	0.002
Treatments		Mean	SE	Mean	SE	Mean	SE	Mean	SE	Mean	SE
HP		1.035	0.052	0.066	0.004	0.962	0.049	0.929	0.003	0.656	0.035
LP		0.876	0.043	0.074	0.004	0.796	0.039	0.908	0.004	0.477	0.026
Mean		0.955	0.035	0.070	0.003	0.879	0.033	0.919	0.003	0.566	0.024
	d.f	AA (mm^2^)		%AA		MXA (mm^2^)		MXV (counts)		WC (m^4^ · 10^−20^)	
		F	P	F	P	F	P	F	P	F	P
Genotype (G)	14	30.346	<0.001	4.225	<0.001	16.118	<0.001	50.071	<0.001	29.836	<0.001
Treatment (P)	1	0.241	0.625	26.059	<0.001	0.010	0.920	0.256	0.614	0.025	0.875
G * P	14	0.712	0.754	1.559	0.118	2.197	0.018	0.605	0.850	1.381	0.191
Treatments		Mean	SE	Mean	SE	Mean	SE	Mean	SE	Mean	SE
HP		0.306	0.018	31.837	0.918	0.00153	0.00008	5.49	0.195	162.209	21.298
LP		0.319	0.020	39.991	1.307	0.00155	0.00008	5.36	0.177	157.718	19.018
Mean		0.313	0.013	35.914	0.904	0.00154	0.00006	5.42	0.131	159.963	14.198

Analysis of variance, means and standard deviation (SE) values are shown for root cross-section area (RXSA), total stele area (TSA) total root cortical area (TCA), proportion of TCA, living cortical area (LCA), aerenchyma area (AA) and percent (%AA), median late metaxylem vessel area (MXA), number of late metaxylem vessels (MXV), and the water conductance (WC) in 15 *O. sativa* accessions evaluated under high (100 μM, HP) and low phosphorus (2 μM, LP) treatments

**Fig. 7:**
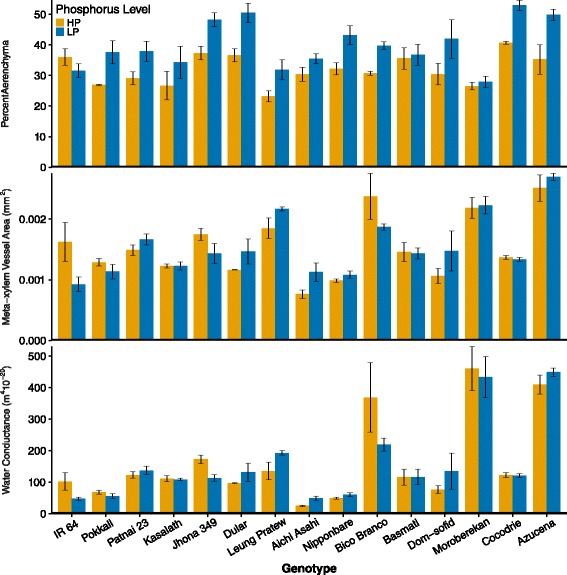
Effects of genotype and P treatment on percent aerenchyma, median metaxylem area and water conductance. Values shown are means of three replications ± SE. See Table 6 for statistical analyses

Significant genotype effects were observed for both absolute area (AA) and percentage (%AA) of aerenchyma (Table 6). Low P availability significantly increased %AA, but not AA. Overall AA was correlated with RXSA and TCA (r > 0.86, *P* < 0.01) but %AA was not correlated with these area traits. Although there was no significant overall genotype x P interaction, the effects of low P on %AA varied from no effect in IR64 and Basmati to an approximately 40 % increase in Azucena, Pokkali and Leung Pratew (Fig. 7). Living cortical area (LCA), the difference between TCA and AA, was reduced by low P as a result of both the increase in %AA and the decrease in TCA (Table 6). *Japonica* genotypes had significantly greater AA, but not %AA and there were no significant varietal group * P interactions for aerenchyma traits (Additional file 3: Table S3).

Significant differences among genotypes were observed for both number (MXV) and median area (MXA) of meta-xylem vessels (Table 6). The *tropical japonica* genotype Moroberekan had the largest number of metaxylem vessels (8.3) while the *temperate japonica* genotype Aichi Asahi had the smallest number (3.8). There was no significant effect of low P on MXV and no significant genotype x P interaction. There was a significant interaction between genotype and P treatment for MXA, with low P effects ranging from a 20 % reduction in Bico Branco to a 48 % increase in Aichi Asahi (Fig. 7). Using the Hagen- Poiseuille equation to convert the number and radii of the xylem vessels to axial water conductance in ideal cylinders, there was a 41 % decrease in axial water conductance in nodal roots of Bico Branco, a 52 % decrease in IR64 (the most negatively affected genotype), and a 98 % increase in axial water conductance in Aichi Asahi (Fig. 7). Varietal groups did not explain any of the differences in P response, since there were no significant varietal group * P interactions for xylem traits (Additional file 3: Table S3).

Allometric relationships for anatomical traits were calculated to determine how each trait scaled with reference to RXSA (Table 5). TCA scaled greater than the isometric value of 0.67 (for area traits) in all subpopulations, indicating that TCA increased disproportionally compared to overall RXSA. The other anatomical variables usually scaled below the isometric value, with a few exceptions including greater than expected scaling exponents for TSA and MXV in *aromatic* and AA in *temperate japonica*.

## Discussion

For most of the root traits measured, we observed both genetic variation in the trait and genetic variation in plasticity of the responses to low P. Genotypic variation was not explained by varietal groups, since there were no significant varietal group by P interactions. Genotypes varied in the extent and sometimes the direction of their responses to low P, depending on the trait. When shoot dry weight of low-P plants was plotted as a function of that of high-P plants, a wide range of vigor under both P levels was evident. The three *aus* genotypes (*Jhona 349*, *Dular* and *Kasalath*) suffered less from low-P stress than other genotypes but were also vigorous under high P (Fig. 3). Under low P availability, *aus* genotypes had greater shoot P content (Fig. 2) and less reduction in length of large and small lateral roots (averaging 6 % reduction in *aus* vs. 15 % overall) than other genotypes. The root to shoot ratio was greater in *aus* than in other genotypes for both P treatments (at high P, 0.36 in *aus* vs. 0.25 for other genotypes, at low P, 0.41 in *aus* vs. 0.31 for other genotypes). The *aus* subpopulation originates from a region in India with infertile and drought-prone soils and is a recognized source of stress-tolerance in rice breeding (Londo et al. 2006; Gowda et al. 2012; Gamuyao et al. 2012). The *aus* genotypes in our study included Kasalath and Dular, which were previously shown to have superior performance under low P (Wissuwa and Ae 2001). Kasalath was used as a tolerant parent in a mapping population identifying QTL associated with P efficiency (Wissuwa et al. 1998) leading to the identification of *Pup1*, a QTL conferring tolerance to low P (Wissuwa et al. 2002). The *Pup1* locus includes *PSTOL1* (phosphorus-starvation tolerance 1), a protein kinase that, when expressed in the low P-intolerant lines Nipponbare and IR64 (which lack a *PSTOL1* allele), increases P uptake, root dry weight and surface area, and early nodal root development, accounting for improved performance under P limitation (Gamuyao et al. 2012). In our experiments, the three *aus* lines had greater shoot and root dry weight than the other genotypes regardless of P treatment (for shoots and roots respectively, mean dry weights were 6.39 and 2.24 g for *aus* vs 4.53 and 1.27 g for all other genotypes). Later work showed that Moroberekan, a *tropical japonica* line, has an *O. glaberrima* allele at the *PSTOL1* locus and Kasalath markers in other sections of the *Pup1* region thought to be important for low-P tolerance (Pariasca-Tanaka et al. 2014). Moroberekan performed very well under low P in our experiments (Fig. 3), but had average root and shoot DW and had the highest increase in root dry weight in response to low P of all genotypes (37 % increase vs. average 23 % increase), in contrast to *aus* lines which had only small (12 %) increases in root dry weight. The mechanisms by which Moroberekan maintains its performance under low P therefore appear distinct from those of the *aus* lines, and could be related to the plasticity of root growth rather than constitutively large roots and a high root to shoot ratio.

Lateral roots in rice are classified into two main types: small and large lateral roots (Rebouillat et al. 2009). Small lateral roots are determinate, remain short and fine and do not support secondary branching, while large lateral roots can become very long and branch further. While P deficiency resulted in reduced length of both classes of lateral roots in this study, the effect on large lateral roots was relatively smaller, resulting in an average of 15 % decrease in the proportion of lateral root length as small lateral roots. The reduction in lateral root elongation is partially explained by the overall decrease in plant growth under low phosphorus, since this would also decrease resources available for continued root development. Indeed, the allometric scaling exponent for small lateral root length was the same for high and low P plants, indicating that the proportional investment in small lateral roots relative to shoot DW was approximately equal (Table 5). Large lateral root density, however, did not have a significant allometric relationship with shoot dry weight, but was also reduced with low phosphorus (Tables 4 and 5).

Although other studies have described increased elongation and density of lateral roots grown under phosphorus stress, e.g. in *Arabidopsis* (Williamson et al. 2001; Linkohr et al. 2002; Lopez-Bucio et al. 2002; Reymond et al. 2006; Niu et al. 2013) and in some maize genotypes (Zhu and Lynch 2004), these studies were conducted with seedlings and, with the exception of the Zhu et al. study (Zhu and Lynch 2004), without phosphorus buffering, which results in progressive P starvation as seed stores are depleted. We cultivated plants to the V8 stage (well beyond the seedling stage) in diffusion-limited phosphorus using solid-phase buffered Al-P (Lynch et al. 1990), which produces realistic phosphorus availability conditions. Consistent with our results, when low P concentrations were maintained by renewing the nutrient solution, lateral root elongation was reduced in maize at the V5–V9 stage, despite an increased root to shoot ratio (Mollier and Pellerin 1999). Buffered low P also reduced lateral root length and density while maintaining basal root growth in common bean (Borch et al. 1999).

The effect of low phosphorus on small vs. large lateral root length and density in rice has not previously been examined. While large lateral roots could be useful to increase the range of soil exploration for patches of greater phosphorus availability, they would also be more costly to construct, since they have larger diameter and length. Recent modeling of maize root systems showed that short, dense lateral roots were superior to long, sparse lateral roots for phosphorus capture (Postma et al. 2014). Genotypes in the *aus* sub-population had relatively greater lengths of both small and large lateral roots, and a greater proportion of small lateral roots under low phosphorus (Fig. 4). These traits could contribute to their overall high shoot dry weight and P content under low phosphorus (Figs. 2 and 3) and to the known P-efficiency of *aus* genotypes. The *aus* genotype Kasalath, the source of the phosphorus uptake 1 (*Pup1*) QTL encompassing the phosphorus-starvation tolerance 1 locus (*PSTOL1*), had the greatest lengths of small and large lateral roots in this study (Fig. 4). Introgression of the *Pup1* QTL and overexpression of *PSTOL1* are associated with greater root surface area and greater P uptake, especially under P-deficiency (Wissuwa 2005; Gamuyao et al. 2012), while down-regulation of *PSTOL1* reduced root number and surface area. Greater length and proportion of small lateral roots may contribute to phosphorus stress tolerance associated with *Pup1*.

The importance of root hair traits for P uptake is well established (Lynch 2011; Brown et al. 2012a), and root hairs are also believed to be important for uptake of other nutrients (Drew and Nye 1969; Gahoonia et al. 2006; Samal et al. 2010), for rhizosheath formation, and for penetration of hard soils (Brown et al. 2012b; Haling et al. 2013; Lynch et al. 2014). It has been suggested that root hairs could make an important contribution to the efficiency of P uptake, which, together with root size, explains a high proportion of the variation in P acquisition and growth for rice (Mori et al. 2016). The rice genotypes tested here demonstrated some genetic variation for root hair length and root hair density (approximately 1.5 and 1.3-fold, respectively), indicating potential for breeding this trait for improved P-acquisition efficiency. Root hair density was positively correlated with yield after seedling-stage drought in direct-seeded upland conditions (Sandhu et al. 2015). Rice root hairs are short compared to other crops. In these experiments, mean root hair length was 0.23 mm, while root hair lengths ranged from 0.32 to 0.83 mm in common bean (Miguel et al. 2015), 0.40–1.34 mm in barley (Gahoonia and Nielsen 2004) and 0.62–4.07 mm in maize (Zhu et al. 2005b). While this leaves plenty of room for improvement, breeding for root hair traits will be challenging since root hair development depends strongly on the growth environment. A recent study showed that rice root hair length and density varied among root classes and with growth medium (Nestler et al. 2016). Root hairs on plants grown in nutrient solution were much longer, denser and more responsive to low P than root hairs on plants grown in soil in either rhizoboxes or in the field (Nestler et al. 2016). In our experiment using buffered P in a sand-vermiculite medium, nodal root hairs were about 30 % longer than root hairs found on main (nodal) roots grown in the greenhouse in a P-fixing Andosol, but about 40 % shorter than root hairs from plants grown in nutrient solution, and we found a 15 % average increase in root hair length with low P compared to 10 % in rhizoboxes with P-fixing Andosol (Nestler et al. 2016). Nester etl al. found that root hair density was increased with low P only in nutrient solution, and was unaffected or reduced by low P in rhizoboxes and an upland field, while we found an average of 10 % increase in root hair density with low P. Clearly, the response of rice root hairs to low P depends strongly on the growth medium, and the use of soil or buffered P provides a more realistic assessment of root hair phenotypes than hydroponic culture.

Tropical rice genotypes within the *Japonica* varietal group have been described as having thick roots, while *Japonica* genotypes from temperate zones (group 6 T) had roots of intermediate thickness (Lafitte et al. 2001). When root diameter, measured as the total root cross-section area (RXSA), was compared across genotypes, we found that the *Japonica* genotypes had about 32 % greater average RXSA than the *Indica* genotypes (Additional file 3: Table S3), but that the two *temperate japonica* genotypes, Aichi Asahi and Nipponbare, had RXSA similar to *Indica* genotypes (Additional file 4: Figure S4). However, there was considerable variation among genotypes within groups and an overlapping distribution between groups. Plasticity of RXSA response to low P varied across genotypes, but most genotypes had thinner roots under low-P stress (Table 6). Reduction of root diameter in response to low P availability, a process termed “root etiolation”, has been suggested to reduce the metabolic cost of soil exploration (Lynch 2011). Consistent with this idea, low P mostly affected RXSA via its effects on the cortical area (TCA), by far the largest component of RXSA (Table 6) and therefore of root volume. All genotypes showed a reduction in TCA with low P, while stele area (TSA) showed variable responses to low P. The metabolic cost of soil exploration is predicted to decline under low P stress via reduction of the total area of the cortex and increasing aerenchyma formation (%AA), which combine to reduce the living cortical area (LCA) (Table 6). Reduced LCA due to greater aerenchyma formation has been shown to reduce specific root respiration in maize, permitting greater root elongation, better resource capture and greater yields under drought and nitrogen limitation in maize (Fan et al. 2003; Zhu et al. 2010; Jaramillo et al. 2013; Saengwilai et al. 2014; Chimungu et al. 2015).

Root cortical aerenchyma (RCA) consists of large air-filled intercellular spaces or lacunae in the root cortex. In rice, the formation of aerenchyma is associated with cell lysis and likely to be coordinated by a programmed cell death (PCD) mechanism (Kawai et al. 1998; Parlanti et al. 2011). Rice, like other species tolerant of soil hypoxia, exhibits strong development of constitutive RCA, which provides a pathway of low resistance to diffusion of atmospheric oxygen to the root tips (Justin and Armstrong 1987; Colmer 2003; Suralta and Yamauchi 2008). Although RCA formation is commonly increased by hypoxia, RCA is also stimulated by a variety of edaphic stresses in maize, including low phosphorus, nitrogen, sulfur, high temperature and drought (Konings and Verschuren 1980; Drew et al. 1989; Kirk and van Du 1997; Przywara and Stepniewski 2000; Bouranis et al. 2003; Evans 2004; Fan et al. 2007; Zhu et al. 2010). In this study, %AA increased by an average of 25 % under low P, indicating that even in the background of high constitutive aerenchyma formation, nutrient deficiency can have additional impact on aerenchyma formation and likely on root metabolic cost.

The effect of low P on stele (TSA) and xylem vessel (MXA) areas depended on the genotype and followed similar patterns as RXSA since these traits were correlated (for RXSA vs. TSA, *r* = 0.83, for TSA vs. MXA, *r* = 0.56), as previously reported (Kondo et al. 2000; Uga et al. 2008). *Aromatic* and *tropical japonica* accessions had larger TSA and MXA than other genotypes (Additional file 3: Figure S3). The variation among genotypes in the response of stele area to low P has not been previously reported. Although TCA and TSA are correlated overall (*r* = 0.81), and many genotypes responded to low P with smaller or unchanged TSA, a few genotypes (notably Azucena and Leung Pratew) responded with increased stele area and reduced TCA. The possible impact of this response is not known.

Genetic variation for root xylem features has been observed in wheat (Richards and Passioura 1981), maize (Burton et al. 2013, 2015; York et al. 2015), and rice (Kondo et al. 2000; Lafitte et al. 2001; Uga et al. 2008). In this study, we found that genotype had a greater effect on metaxylem vessel area (MXA) and number (MXV) than phosphorus treatment, which did not affect MXV (Table 6). The *tropical japonica* and *aromatic* accessions had larger median area (MXA) and greater number (MXV) of meta-xylem vessels, both of which correlated with the larger root diameter and TSA. MXA and MXV were used to calculate the potential water conductance at several axial positions along the nodal root, revealing genetic variation for water conductance distribution as well as for maximal water conductance (Fig. 1). While most genotypes showed maximal conductance at the middle positions of the root, five genotypes showed maximal conductance at the position closest to the root tip (5 cm). All genotypes showed relatively restricted water conductance at or near the base of the root. This would be expected to help maintain water potential within the root to permit continued growth and function even as soil water potentials drop.

Low P has been shown to reduce root axial hydraulic conductivity in cotton (Radin and Eidenbock, 1984), though this was not specifically linked to xylem architecture, and later work suggested that nutrient-deprivation induced changes in root hydraulic conductivity were physiological and related to radial conductance rather than structural and related to axial conductance (Radin 1990; Clarkson et al. 2000). Our calculations of predicted water conductance at a single sampling point on a representative nodal root did not reveal consistent low-P effects, since only three of the 15 genotypes (IR64, Jhona 349, Bico Branco) had significantly lower predicted water conductivity with low P (data not shown). More detailed assessment of xylem traits among root classes and along the root axis is needed to determine the role of xylem anatomy in water conductance.

## Conclusions

Development of new cultivars of rice and other cereals that are more productive under low phosphorus is an important priority for agriculture (Lynch 2011; Vinod and Heuer 2012; Rose et al. 2012; Brown et al. 2012a). Our results demonstrate significant variation for root traits predicted to influence phosphorus efficiency in rice. In addition, we demonstrate plasticity of many root traits in response to low phosphorus, an important characteristic to consider when breeding for low phosphorus adaptation in crops. There was genetic variation in plasticity for many traits. For most traits, with the exception of stele area and meta-xylem vessel area, the direction of low-P response was uniform among genotypes, and in every case the genotype by phosphorus interactions were small (*F* values were small compared to main effects). Therefore, evaluation of root phenotypes from plants grown under fertile conditions should be adequate for genetic mapping and experiments where assessment under low-P conditions is difficult.

## Methods

### Plant Cultivation and Phosphorus Treatments

The experiments were carried out in a greenhouse located on the campus of the Pennsylvania State University, University Park, PA (40°48′ N, −77°51′ W). The study employed 15 *O. sativa* accessions (Table 1) selected from the publically available Rice Diversity Panel 1 (Eizenga et al. 2013) and provided by Susan McCouch, Cornell University. Genotypes were chosen from each of the five sub-populations and two varietal groups based on popularity for genetic and physiological studies. Three replications were grown per genotype, and replications were staggered in time. Rice seeds were surface sterilized with 10 % bleach and pre-germinated prior planting. For pre-germination, seeds were sown on moist paper towel soaked with 0.5 mM CaSO_4_ for 72 h at 28 °C in an incubator. Healthy germinated seeds were transplanted to 10.5 L pots (21 × 40.6 cm, top diameter × height, Nursery Supplies Inc., Chambersburg, PA, USA) filled with a mixture (volume based) of 40 % medium size (0.3–0.5 mm) commercial grade sand (Quikrete Companies Inc., Harrisburg, PA, USA), 60 % horticultural vermiculite (Whittemore Companies Inc.), and 1 % solid-phase buffered P (Al-P, prepared according to Lynch et al. 1990) providing a constant availability of low (2 μM) or sufficient (100 μM) P in the soil solution. Plants were irrigated once per day with P-free Yoshida nutrient solution (Yoshida et al. 1971) via drip irrigation.

### Root and Shoot Growth Measurements

Plants were harvested at the 8th leaf stage (V8), when the roots were just beginning to reach the bottom of the pots. At harvest, the number of tillers and shoot height were recorded. Root systems were excavated, washed and preserved in 70 % ethanol until the time of processing and analysis. Shoots and roots were dried at 65 °C for 72 h prior to dry weight determination.

### Root Architecture Measurements

The number of nodal (crown) roots were counted for each root system. A representative 30-cm-long nodal root was collected from each plant to assess small and large lateral root length and lateral root density. The nodal roots were scanned using a flatbed scanner at a resolution of 600 dpi (HP ScanJet II, Hewlett Packard, USA). Root analysis software *WinRhizo* (Regent Instruments, Quebec, Canada) was used to determine lateral root branching, which was categorized using two diameter classes of < 0.10 mm (small lateral roots) and 0.10–0.70 mm (large lateral roots). Lateral root number per nodal root (lateral root density) was counted manually from the scanned image.

### Root Hair Measurements

For the axial distribution study, root hair length and density (number of root hairs per mm^2^ root surface area) were observed on approximately 20-cm-long nodal roots, at 5, 10, 15 cm from the root tip. For assessment of the effects of phosphorus on root hair length and density, root hairs were measured on a root segment taken 5–10 cm from the root tip. Roots were instantaneously immersed in 0.5 % Toluidine Blue to permit clear observation of root hairs (Additional file 5: Figure S5). The stained roots were observed at 40× magnification with a dissecting microscope (SMZ-U, Nikon, Tokyo, Japan), equipped with a digital camera (NIKON DS-Fi1, Tokyo, Japan). Separate images for length and density were captured for each sample, focusing on the root edge and center respectively. *Image J* software (National Institute of Mental Health, Bethesda, Maryland, USA.) was used for quantitative analysis of root hair length and density.

### Measurement of Root Anatomical Traits

For the axial distribution study, root anatomical structures were observed on 20-cm-long nodal roots, at 5, 10 and 15 cm from the root tip and at 1 cm from the basal end of the root. In the low P study, samples for anatomical analysis were collected 10–15 cm from the nodal root tip, based on maximum development of aerenchyma at that position as determined in the distribution study. Preserved roots were freehand-sectioned using Teflon-coated double-edged stainless steel blades (Electron Microscopy Sciences, Hatfield, PA, USA) and stained with 0.5 % Toluidine Blue. Transverse sections were examined under a Diaphot inverted light microscope (Nikon, Chiyoda-ku, Japan). The three best root cross-sections were selected and images captured with a black and white XC-77 CCD Video Camera (Hamamatsu, Iwata-City, Japan). The image analysis software *RootScan* (Burton et al. 2012b) was used for quantitative analysis of total root cross-sectional area (RXSA), cortical area (TCA), aerenchyma area (AA), stele area (TSA), number of late metaxylem vessels (MXV), median metaxylem vessel area (MXA). Percent RCA (%AA) was calculated from AA as a proportion of TCA. Living cortical area (LCA) was calculated as the difference between TCA and AA. The water conductance parameter (WCP) is the sum of individual conductances of the late metaxylem vessels, calculated (as ideal cylinders) using the Hagen-Poiseuille equation (WCP = π•r^4^).

### Tissue P Content

Dry samples of root and shoot tissue were ground and ashed at 495 °C for 12 h. The ash was dissolved by adding 8 mL of 100 mM HCl and analyzed for P concentration spectrophotometrically according to the Murphy and Riley method (Murphy and Riley 1962).

### Experimental Design and Data Analysis

A randomized complete block design was used with the time of planting as a block effect. Statistical analyses were performed using package R, version 3.0.2 (R Foundation for Statistical Computing, Vienna, Austria) and Minitab version 16.2 (Minitab Inc., University Park, USA). For the distribution study, root hair and anatomical trait distribution data was analyzed with 1- and 2-way analyses of variance (ANOVA) to determine the main effects and interactions between genotype and sampling position. Low P treatments were not included in the sampling position study. One- and two-way analyses of variance (ANOVA) were used to examine the influence of P treatments on dependent variables and interactions between genotype (G) and P level (G × P). Reported correlation values are Pearson Correlation coefficients (r).

### Allometric Analyses

Allometric analyses were performed by calculating the linear regression of the natural logarithm of the total plant dry weight as a function of the natural logarithms of small and large lateral root lengths and lateral root density, or for root anatomical traits, by calculating the linear regression of the natural logarithm of the root cross-sectional area as a function of the natural logarithms of each of the anatomical traits. The coefficient of determination (R^2^) and the slope of the regression line (α) were recorded.

## Abbreviations

%AA, percent aerenchyma area as a proportion of total cortical area; AA, aerenchyma area; MXA, median late metaxylem vessel area; MXV, number of late metaxylem vessels; RXSA, total root cross-sectional area; TCA, total cortical area; TSA, total stele area; WC, water conductance

## Additional files

Additional file 1: Figure S1. Distribution of total stele area and median late metaxylem vessel area by genotype and axial position. Samples were taken close to the base and at 5, 10 or 15 cm from a nodal root tip. Values shown are means of three replications. See Table 3 for statistical analyses. (PDF 9 kb) Additional file 2: Table S2. The effects of varietal group and phosphorus treatment on shoot and root morphological traits. Analysis of variance (ANOVA), means and standard error (SE) values are shown for 7 *Indica* and 8 *Japonica* genotypes grown with high (100 μM; HP) or low phosphorus (2 μM; LP) treatments. (DOCX 134 kb) Additional file 3: Table S3. The effects of varietal group and phosphorus treatment on anatomical traits. Analysis of variance, means and standard deviation (SE) values are shown for root cross-section area (RXSA), total stele area (TSA) total root cortical area (TCA), proportion of TCA, living cortical area (LCA), aerenchyma area (AA) and percent (%AA), median late metaxylem vessel area (MXA), number of late metaxylem vessels (MXV), and the water conductance (WC) for 7 *Indica* and 8 *Japonica* genotypes evaluated under high (100 μM, HP) and low phosphorus (2 μM, LP) treatments. (DOCX 132 kb) Additional file 4: Figure S4. Effects of genotype and phosphorus treatment on root cross-sectional area. Values shown are means of three replications ± SE. See Table 6 for statistical analyses. (PDF 6 kb) Additional file 5: Figure S5. Quantitative analysis of root hair density (left) and root hair length (right). Roots were stained with Toluidine Blue dye and root hairs were measured on 20-25 cm-long nodal roots using ImageJ software. (PDF 1495 kb)
