# Supplementary material for: Genetic Variability in Phosphorus Responses of Rice Root Phenotypes
Source: Rice (N Y). 2016 Jun 13;9:29. doi: 10.1186/s12284-016-0102-9 (PMC4905936; doi:10.1186/s12284-016-0102-9)
Supplement: Additional file 1: Figure S1. — Distribution of total stele area and median late metaxylem vessel area by genotype and axial position. Samples were taken close to the base and at 5, 10 or 15 cm from a nodal root tip. Values shown are means of three replications. See Table 3 for statistical analyses. (PDF 9 kb) [file 12284_2016_102_MOESM1_ESM.pdf]

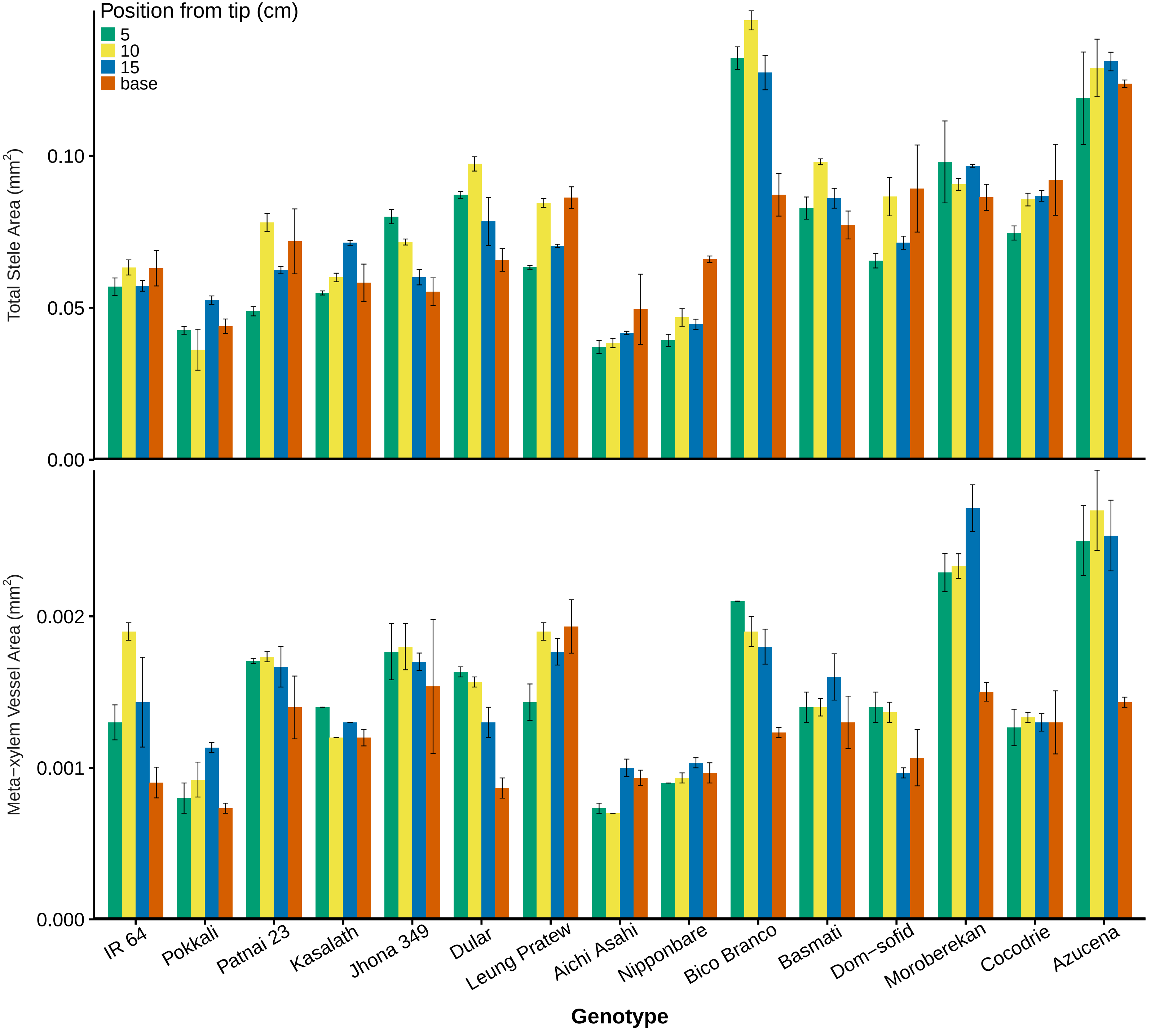

Position from tip (cm)
5
10
15
base
Total Stele Area (mm^2)
0.00
0.05
0.10
Meta−xylem Vessel Area (mm^2)
0.000
0.001
0.002
IR 64
Pokkali
Patnai 23
Kasalath
Jhona 349
Dular
Leung Pratew
Aichi Asahi
Nipponbare
Bico Branco
Basmati
Dom−sofid
Moroberekan
Cocodrie
Azucena
Genotype
