# Supplementary material for: Genetic Variability in Phosphorus Responses of Rice Root Phenotypes
Source: Rice (N Y). 2016 Jun 13;9:29. doi: 10.1186/s12284-016-0102-9 (PMC4905936; doi:10.1186/s12284-016-0102-9)
Supplement: Additional file 2: Table S2. — The effects of varietal group and phosphorus treatment on shoot and root morphological traits. Analysis of variance (ANOVA), means and standard error (SE) values are shown for 7 Indica and 8 Japonica genotypes grown with high (100 μM; HP) or low phosphorus (2 μM; LP) treatments. (DOCX 134 kb) [file 12284_2016_102_MOESM2_ESM.docx]

**Table S2. The effects of varietal group and** **phosphorus treatment on shoot and root morphological traits**. Analysis of variance (ANOVA), means and standard error (SE) values are shown for 7 *Indica* and 8 *Japonica* genotypes grown with high (100 µM; HP) or low phosphorus (2 µM; LP) treatments.

|  | d.f. | Shoot biomass  (g·plant^-1^) | | Plant height  (cm) | | Number of tillers  (plant^-1^) | | Root dry weight  (g·plant^-1^) | |
| --- | --- | --- | --- | --- | --- | --- | --- | --- | --- |
|  |  | **F** | **P** | **F** | **P** | **F** | **P** | **F** | **P** |
| Varietal group (V) | 1 | 0.825 | 0.366 | 10.838 | 0.001 | 22.612 | <0.001 | 5.752 | 0.019 |
| Treatment (P) | 1 | 82.269 | <0.001 | 9.979 | 0.002 | 33.657 | <0.001 | 1.874 | 0.175 |
| V * P | 1 | 0.343 | 0.560 | 0.011 | 0.915 | 2.731 | 0.102 | 0.101 | 0.751 |
| **Varietal group** |  | **Mean** | **SE** | **Mean** | **SE** | **Mean** | **SE** | **Mean** | **SE** |
| *Indica* |  | 4.74 | 0.249 | 100.31 | 2.113 | 3.60 | 0.180 | 1.27 | 0.099 |
| *Japonica* |  | 5.10 | 0.317 | 90.98 | 1.831 | 5.60 | 0.398 | 1.68 | 0.143 |
| **Mean** |  | 4.91 | 0.199 | 95.96 | 1.491 | 4.53 | 0.233 | 1.46 | 0.087 |
|  | d.f | Root hair length  (mm) | | Root hair density  (hairs·mm^-2^) | | Nodal root number(plant^-1^) | | Root:shoot ratio | |
|  |  | **F** | **P** | **F** | **P** | **F** | **P** | **F** | **P** |
| Varietal group (V) | 1 | 45.718 | <0.001 | 8.847 | 0.004 | 3.125 | 0.081 | 3.246 | 0.075 |
| Treatment (P) | 1 | 26.228 | <0.001 | 47.354 | <0.001 | 110.074 | <0.001 | 14.060 | <0.001 |
| V * P | 1 | 0.076 | 0.783 | 0.959 | 0.330 | 0.136 | 0.713 | 0.006 | 0.941 |
| **Varietal group** |  | **Mean** | **SE** | **Mean** | **SE** | **Mean** | **SE** | **Mean** | **SE** |
| *Japonica* |  | 0.211 | 0.005 | 231.76 | 2.559 | 70.21 | 2.61 | 0.278 | 0.019 |
| *Indica* |  | 0.257 | 0.005 | 219.42 | 3.335 | 77.67 | 3.39 | 0.331 | 0.022 |
| **Mean** |  | 0.232 | 0.004 | 226.01 | 2.159 | 73.69 | 2.13 | 0.303 | 0.015 |
|  | d.f | Small lateral  root length (cm) | | Large lateral  root length (cm) | | Lateral root density | | Shoot P content  (mg·plant^-1^) | |
|  |  | **F** | **P** | **F** | **P** | **F** | **P** | **F** | **P** |
| Varietal group (V) | 1 | 4.902 | 0.029 | 1.462 | 0.230 | 3.864 | 0.052 | 0.362 | 0.549 |
| Treatment (P) | 1 | 37.796 | <0.001 | 67.354 | <0.001 | 85.971 | <0.001 | 142.801 | <0.001 |
| V * P | 1 | 0.078 | 0.781 | 0.018 | 0.892 | 0.148 | 0.701 | 0.390 | 0.534 |
| **Varietal group** |  | **Mean** | **SE** | **Mean** | **SE** | **Mean** | **SE** | **Mean** | **SE** |
| *Japonica* |  | 62.39 | 5.118 | 179.78 | 10.09 | 44.44 | 1.34 | 9.14 | 0.903 |
| *Indica* |  | 81.52 | 7.155 | 198.39 | 11.73 | 40.79 | 1.26 | 9.95 | 0.988 |
| **Mean** |  | 71.31 | 4.405 | 188.46 | 7.70 | 42.73 | 0.94 | 9.52 | 0.665 |
