# Supplementary material for: Genetic Variability in Phosphorus Responses of Rice Root Phenotypes
Source: Rice (N Y). 2016 Jun 13;9:29. doi: 10.1186/s12284-016-0102-9 (PMC4905936; doi:10.1186/s12284-016-0102-9)
Supplement: Additional file 3: Table S3. — The effects of varietal group and phosphorus treatment on anatomical traits. Analysis of variance, means and standard deviation (SE) values are shown for root cross-section area (RXSA), total stele area (TSA) total root cortical area (TCA), proportion of TCA, living cortical area (LCA), aerenchyma area (AA) and percent (%AA), median late metaxylem vessel area (MXA), number of late metaxylem vessels (MXV), and the water conductance (WC) for 7 Indica and 8 Japonica genotypes evaluated under high (100 μM, HP) and low phosphorus (2 μM, LP) treatments. (DOCX 132 kb) [file 12284_2016_102_MOESM3_ESM.docx]

**Table S3.** **The effects of varietal group and phosphorus treatment on** **anatomical traits**. Analysis of variance, means and standard deviation (SE) values are shown for root cross-section area (RXSA), total stele area (TSA) total root cortical area (TCA), proportion of TCA, living cortical area (LCA), aerenchyma area (AA) and percent (%AA), median late metaxylem vessel area (MXA), number of late metaxylem vessels (MXV), and the water conductance (WC) for 7 *Indica* and 8 *Japonica* genotypes evaluated under high (100 µM, HP) and low phosphorus (2 µM, LP) treatments.

|  | d.f. | RXSA (mm^2^) | | TSA (mm^2^) | | TCA (mm^2^) | | TCA:RXSA Ratio | | LCA (mm^2^) | |
| --- | --- | --- | --- | --- | --- | --- | --- | --- | --- | --- | --- |
|  |  | **F** | **P** | **F** | **P** | **F** | **P** | **F** | **P** | **F** | **P** |
| Varietal group (V) | 1 | 17.358 | <0.001 | 12.469 | 0.001 | 17.500 | <0.001 | 4.427 | 0.038 | 10.241 | 0.002 |
| Treatment (P) | 1 | 5.564 | 0.021 | 1.886 | 0.173 | 6.996 | 0.010 | 21.756 | <0.001 | 17.178 | <0.001 |
| V * P | 1 | 0.100 | 0.752 | 0.014 | 0.906 | 0.113 | 0.737 | 0.406 | 0.526 | 0.273 | 0.603 |
| **Varietal group** |  | **Mean** | **SE** | **Mean** | **SE** | **Mean** | **SE** | **Mean** | **SE** | **Mean** | **SE** |
| *Japonica* |  | 1.078 | 0.055 | 0.078 | 0.005 | 0.996 | 0.052 | 0.924 | 0.003 | 0.634 | 0.037 |
| *Indica* |  | 0.814 | 0.024 | 0.060 | 0.002 | 0.746 | 0.024 | 0.913 | 0.004 | 0.490 | 0.022 |
| **Mean** |  | 0.955 | 0.035 | 0.070 | 0.003 | 0.879 | 0.033 | 0.919 | 0.003 | 0.566 | 0.024 |
|  | d.f | AA (mm^2^) | | %AA | | MXA (mm^2^) | | MXV (counts) | | WC (m^4^· 10^-20^) | |
|  |  | **F** | **P** | **F** | **P** | **F** | **P** | **F** | **P** | **F** | **P** |
| Varietal group (V) | 1 | 19.909 | <0.001 | 1.262 | 0.264 | 2.142 | 0.147 | 14.220 | <0.001 | 10.211 | 0.002 |
| Treatment (P) | 1 | 0.241 | 0.625 | 26.059 | <0.001 | 0.010 | 0.920 | 0.256 | 0.614 | 0.025 | 0.875 |
| V * P | 1 | 0.007 | 0.935 | 0.006 | 0.941 | 0.289 | 0.592 | 0.105 | 0.746 | 0.002 | 0.961 |
| **Varietal group** |  | **Mean** | **SE** | **Mean** | **SE** | **Mean** | **SE** | **Mean** | **SE** | **Mean** | **SE** |
| *Japonica* |  | 0.363 | 0.020 | 36.86 | 1.22 | 0.00162 | 0.00009 | 5.85 | 0.186 | 200.36 | 21.60 |
| *Indica* |  | 0.256 | 0.011 | 34.83 | 1.34 | 0.00145 | 0.00006 | 4.93 | 0.154 | 113.79 | 42.80 |
| **Mean** |  | 0.313 | 0.013 | 35.91 | 0.90 | 0.00154 | 0.00006 | 5.42 | 0.131 | 159.96 | 21.60 |
