# Supplementary figures and images for: Genetic Variability in Phosphorus Responses of Rice Root Phenotypes
Source: Rice (N Y). 2016 Jun 13;9:29. doi: 10.1186/s12284-016-0102-9 (PMC4905936; doi:10.1186/s12284-016-0102-9)

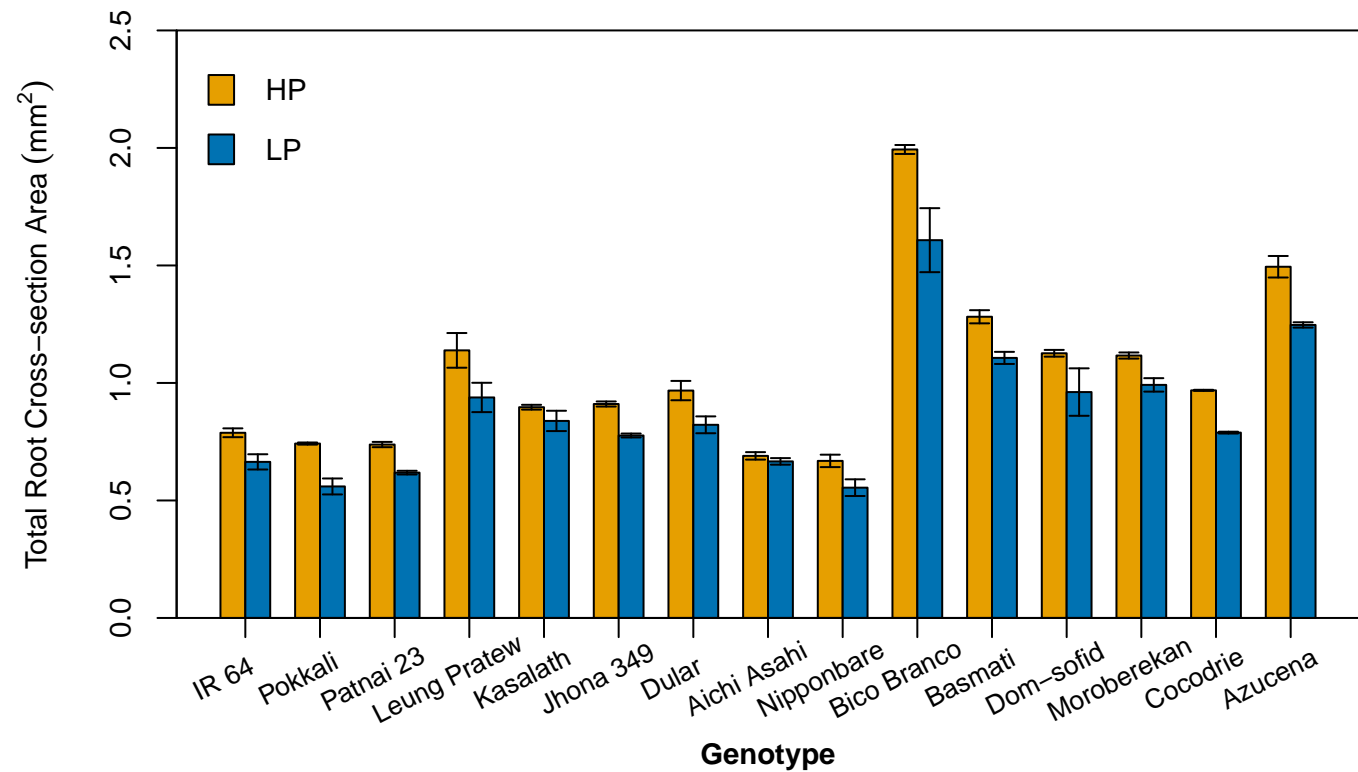

Supplement: Additional file 4: Figure S4. — Effects of genotype and phosphorus treatment on root cross-sectional area. Values shown are means of three replications ± SE. See Table 6 for statistical analyses. (PDF 6 kb) [file 12284_2016_102_MOESM4_ESM.pdf]

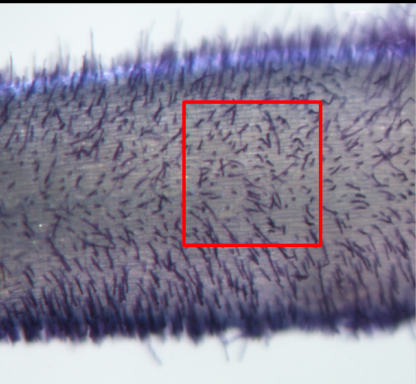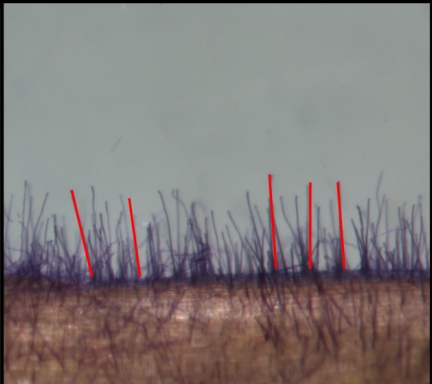

Supplement: Additional file 5: Figure S5. — Quantitative analysis of root hair density (left) and root hair length (right). Roots were stained with Toluidine Blue dye and root hairs were measured on 20-25 cm-long nodal roots using ImageJ software. (PDF 1495 kb) [file 12284_2016_102_MOESM5_ESM.pdf]
